# Association of mid-regional pro-adrenomedullin with office and 24-h ambulatory blood pressure in a Swiss general population sample

**DOI:** 10.1097/HJH.0000000000003866

**Published:** 2024-09-18

**Authors:** Julia Baldwin, Michel Burnier, Belen Ponte, Daniel Ackermann, Menno Pruijm, Bruno Vogt, Murielle Bochud

**Affiliations:** a Department of Epidemiology and Health Systems, Unisanté; b Faculty of Biology and Medicine, University of Lausanne; c Department of Nephrology, University Hospital of Geneva (HUG); d Department of Nephrology and Hypertension, Inselspital, Bern University Hospital and University of Bern; e Service of Nephrology and Hypertension, Centre Hospitalier Universitaire Vaudois and University of Lausanne, Switzerland

**Keywords:** adrenomedullin, blood pressure, blood pressure monitoring ambulatory, cohort studies, pulse pressure

## Abstract

**Objective:**

Adrenomedullin (ADM) is a potent vasodilator. The association between plasma ADM levels and blood pressure (BP) remains unclear. We assessed the association between mid-regional-pro-ADM (MR-proADM) and BP in a multicenter population- and family-based cohort.

**Methods:**

We used data from the Swiss Kidney Project on Genes in Hypertension (SKIPOGH). We included participants present at both baseline and 3-year follow-up (*N* = 843). We examined the association of baseline MR-proADM with baseline office and 24 h ambulatory BP as well as the 3-year change in office BP. In secondary analyses, we studied the association between baseline MR-proADM and 3-year changes in pulse wave velocity (PWV), renal resistive index (RRI) and augmentation index (AI). Mixed-effects linear regression models were used.

**Results:**

In cross-sectional analyses, MR-proADM was negatively associated with office, 24-h and daytime diastolic BP (DBP). MR-proADM was positively associated with nighttime systolic BP (SBP). In longitudinal analyses, baseline MR-proADM was associated with an increase in office SBP and pulse pressure (PP) over 3 years [β (95% CI): 8.2 (0.4, 15.9) and β (95% CI): 6.4 (0.3, 12.4), respectively] but not with changes in PWV, RRI and AI.

**Conclusions:**

The cross-sectional negative association of MR-proADM with DBP is in line with known vasodilatory properties of ADM. The positive association between MR-proADM and nighttime SBP at baseline may reflect endothelial dysfunction believed to be part of the pathogenesis of nocturnal hypertension. The association of higher baseline MR-proADM levels with increased SBP and PP at 3-year follow-up suggests that ADM levels could be a marker of cardiovascular risk.

## Graphical abstract

**Figure:**
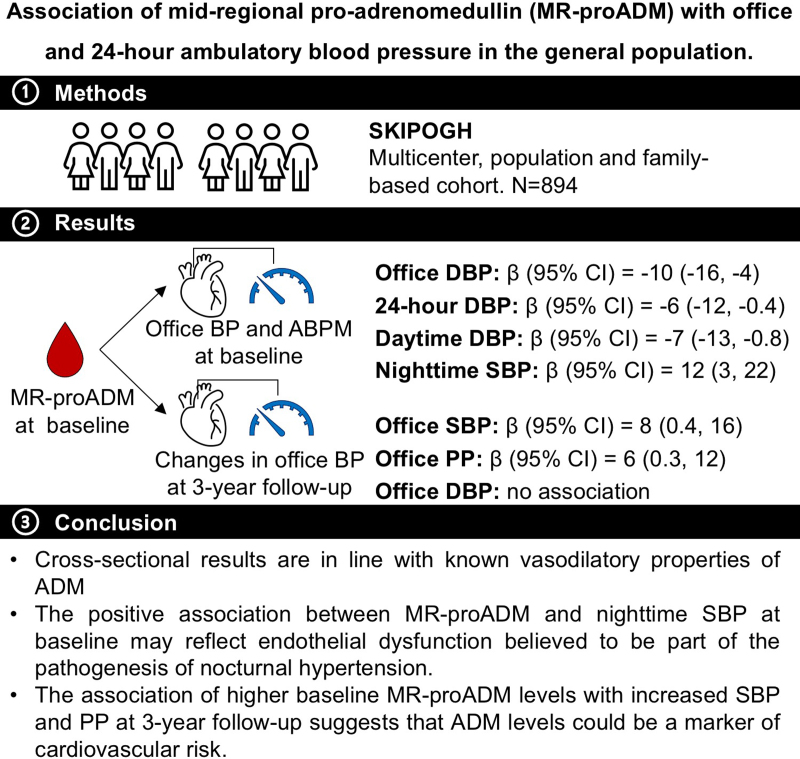

## INTRODUCTION

Adrenomedullin (ADM) is a 52-amino acid peptide synthesized in endothelial and vascular smooth muscle cells that functions as a potent vasodilator. It induces vasodilatation through endothelium-dependent and endothelium-independent mechanisms, including activation of endothelial nitric oxide (NO) synthase [1,2]. In animal models, ADM has also been shown to increase cardiac output not only by decreasing afterload but also through a positive inotrope effect [3–5]. Regulation of ADM is complex. The degradation of ADM is in part regulated by neprilysin; neprilysin cleaves ADM rending the peptide inactive [6]. Concordantly, in patients with chronic heart failure, administration of the neprilysin inhibitor sacubitril/valsartan leads to a significant elevation in mid-regional pro-adrenomedullin (MR-proADM). This proportional increase of ADM markedly exceeds the increase in B-type natriuretic peptide (BNP) and N terminal pro BNP (NT-proBNP). It should be noted that sole administration of valsartan does not have an effect on levels of MR-proADM [7,8]. Physiological stressors such as hypoxia, inflammation and shear stress promote ADM release in animal models [2]. Continuous intra-venous infusion of ADM in humans leads to a decrease in BP, as well as an increase in cardiac output and heart rate [9,10].

Of note, direct measurement of the active peptide ADM, is not considered to accurately quantify overall ADM secretion. In fact, circulating ADM has a short half-life (20-25 min) and binds to complement factor H, which interferes with its quantification [11]. Determination of the mid-regional fragment of pro-ADM (MR-proADM), a preproduct of ADM secreted in equimolar amounts to ADM, is now widely used as a surrogate for ADM secretion as it monitors plasma ADM levels and their changes more accurately. This has been validated in healthy individuals and in patients with septic shock [12,13].

(MR-pro)ADM levels are increased in cardiovascular disease (CVD) and hypertension [14]. This is thought to represent a protective response to increased vascular inflammation, endothelial dysfunction and vascular resistance [15]. Therefore, MR-proADM could be an important readily measurable serum marker of early cardiovascular dysfunction.

A genome-wide association study (GWAS), conducted on 200 000 individuals of European descent, identified the ADM locus as being significantly associated with BP [16]. The direction of this association remains unclear as ADM plasma concentrations were not measured. In a subsequent study, the same locus was associated with ADM gene expression in circulating monocytes and with MR-proADM levels [17]. While these data suggest a causal role of ADM on vascular tone, its precise role in long-term BP control remains unclear.

Diverging results regarding the direction, effect size and presence of an association between MR-proADM and systolic BP (SBP), diastolic BP (DBP), pulse pressure (PP) and pulse wave velocity (PWV) have been published [18–35].

To the best of our knowledge, only one longitudinal study investigating the association between continuous SBP and MR-proADM [35] and one study examining the association between incidence of hypertension and MR-proADM has been published. No study has examined the longitudinal association between MR-proADM and DBP or PP. Therefore, we studied the association between MR-proADM and BP, as well as arterial stiffness, in a multicentric population and family-based cohort, using a cross-sectional and longitudinal model.

## METHODS

### Study population

The Swiss Kidney Project on Genes in Hypertension (SKIPOGH) is a multicenter population and family-based cohort study. Detailed methods have been previously described [36]. The inclusion criteria were as follows: (a) minimum age of 18 years; (b) European descent (defined as having both parents and grandparents born in a restricted list of countries); and (c) at least one first-degree family member willing to participate in the study. Recruitment took place from December 2009 to April 2013. Follow-up visit was conducted from October 2012 to December 2016 on 87% of baseline participants. Written informed consent was obtained from all the participants. The protocol was approved by the Ethics Committee of all the participating universities, namely the Human Research Ethics Committee, Lausanne University Hospital (Lausanne, Switzerland); the Ethics Committee for Research on Human Beings, Geneva University Hospitals (Geneva, Switzerland) and the Ethics Committee of the Canton Bern, (Bern, Switzerland).

### BP measurement

Office BP was measured using a validated nonmercury auscultatory sphygmomanometer (A&D UM-101, A&D Company, Ltd, Toshima Ku, Tokyo, Japan), and internally validated by the SKIPOGH group [37]. This device has met the validation standards of the International Protocol for blood pressure measuring devices established by the ESH and has also undergone internal validation by our research team [38]. After a 10-min rest in the sitting position, BP was measured in each arm. Five measurements were taken on the side with the highest BP [39]. The mean of the last four measures was retained, discarding the first one. Hypertension was defined according to ESH recommendations for office BP [39], that is SBP ≥140 mmHg or DBP ≥90 mmHg and/or use of antihypertensive medication.

24-h ABPM were obtained using a validated Diasys Integra device (Novacor, Rueil-Malmaison, France), with measurements taken at 15-min intervals from 7 a.m. to 10 p.m. and every 30 min thereafter [40]. Day and night periods were obtained from the participants’ 24-h diaries (awake vs. asleep). Monitoring was considered insufficient if <14 SBP and DBP measurements per day were recorded and <7 at night, in accordance with ESH recommendations [39]. Invalid measurements included SBP >280 mmHg or <60 mmHg, DBP >200 mmHg or <40 mmHg, heart rate >200 bpm or <40 bpm and DBP > SBP. Validated measurements were then used to calculate the mean BP.

Office and ABPM PP was calculated as SBP minus DBP.

### Renal Doppler ultrasound

In each study center, the same experienced operator performed renal duplex ultrasounds according to a standardized procedure [36]. RRI were measured in segmental arteries of each kidney. The mean value was multiplied by 100 to express RRI as a percentage.

#### Arterial waveform measurement

In each study center, the same experienced operator obtained arterial waveforms and recorded PWV [41], with the participant in the supine position after a 15-min rest, at the carotid and femoral arteries by applanation tonometry, using a high-fidelity SPC-301 micromanometer (Millar Instruments, Inc., Houston, TX, USA) interfaced with a computer running the SphygmoCor software version 8.0 or 8.2 (AtCor Medical Pty. Ltd, West Ryde, Australia). A validated transfer function was used to obtain central augmentation index (AI) adjusted for heart frequency and central augmented pressure (AP). AP is the difference between second and first systolic peaks. AI is the ratio of AP to aortic PP.

### Mid-regional pro-adrenomedullin

MR-proADM was measured in −80°C frozen EDTA-plasma samples using Time-Resolved Amplified Cryptate Emission (TRACE) technology (B.R.A.H.M.S. KRYPTOR COMPACT Plus, Thermo Fisher Scientific, Hennigsdorf, Germany). The lower detection limit was 0.05 nmol/l, and the functional assay sensitivity (20% inter-assay coefficient of variation) was 0.25 nmol/l. None of the participants had levels below the detection limit.

### Other laboratory measurements

Creatinine and blood glucose were measured in local laboratories using standard clinical laboratory methods.

### Assessment of other covariables

Body mass index (BMI) was calculated by dividing weight by height squared. Glomerular filtration rate was estimated (eGFR) using the 2009 CKD-EPI formula. Chronic kidney disease at baseline was defined as GFR <60 ml/min/1.73 m^2^. Diabetes was defined as fasting blood glucose ≥7 mmol/l, use of hypoglycemic medication and/or self-reported diabetes in questionnaire.

### Statistical analysis

Statistical analyses were conducted using Stata, 17.0 (StataCorp, College Station, TX, USA). Continuous variables were expressed with mean and standard deviation (SD); in case of asymmetric distribution with median and interquartile range (IQR). Categorical variables were described as frequencies and percentages.

To better understand the effect of missing data, characteristics of included and excluded participants were described.

As SKIPOGH is a family-based cohort, we used mixed-effects linear regression model, with family as a random effect term to take into account the correlated nature of the data. In cross-sectional analyses, models were constructed with office or ABPM SBP, DBP, and PP as the dependent variables, taken one-at-a-time, and MR-proADM as the explanatory variable of interest. Models were further adjusted for variables which are thought to be causally associated to both MR-proADM and BP. Model 1 was adjusted for age (centered), sex and study center. For DBP and PP, models were also adjusted for age squared (centered). Model 2 was further adjusted for antihypertensive medication (0, No; 1, Yes), BMI and eGFR. Model 3 was further adjusted for use of a lipid-lowering treatment (0, No; 1, Yes), diabetes (0, No; 1, Yes), smoking status (0, Active smoker; 1, Nonsmoker), and units of alcohol per week.

In the longitudinal analyses, models were constructed with the difference between baseline and follow-up (Δ) office SBP (ΔSBP), office DBP (ΔDBP), and office PP (ΔPP) as dependent variables, and MR-proADM as the explanatory variable. Model 1 was adjusted for age (centered), sex and study center. When DBP was used as the dependent variable, models were further adjusted for age squared (centered). Model 2 was further adjusted for baseline BP (SBP, DBP, or PP for models with ΔSBP, ΔDBP, ΔPP as outcomes, respectively), baseline antihypertensive medication (0, No; 1, Yes), BMI at baseline, Δ weight, ΔeGFR and presence of baseline CKD. Model 3 was further adjusted for use of a lipid-lowering treatment at baseline (0, No; 1, Yes), diabetes (0, No; 1, Yes), smoking status (0, Active smoker; 1, Nonsmoker), and units of alcohol consumed per week.

Secondary analyses were conducted using PWV, RRI, and AI. Models were constructed as described above, except for additional adjustment by baseline SBP, DBP, and heart rate in models 2 and 3. When RRI was used as a dependent variable, models were further adjusted for age squared (centered).

Homogeneity of variance and normality of residuals was assessed for each model. Results of these models were presented as β-coefficients, 95% confidence intervals and *P*-values, the latter being derived from the maximum likelihood ratio test.

As ascertainment of antihypertensive medication via a self-reported questionnaire can lead to errors, we conducted sensitivity analyses excluding (a) hypertensive participants at baseline (and therefore participants taking antihypertensive medication) and (b) both hypertensive and diabetic participants at baseline (c) individuals with untreated hypertension at baseline (d) individuals that started or stopped antihypertensive medication during follow-up.

### Data availability

The data supporting the findings of this study are available from the corresponding author upon reasonable request.

## RESULTS

### Baseline and follow-up characteristics

**FIGURE 1:**
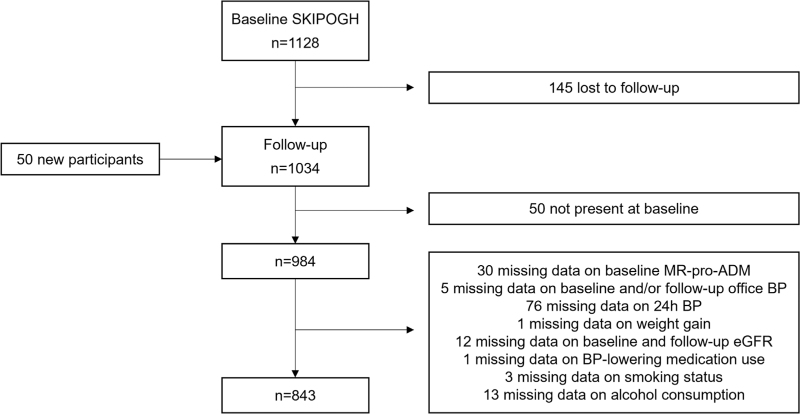
Flow chart of study participants.

**FIGURE 2:**
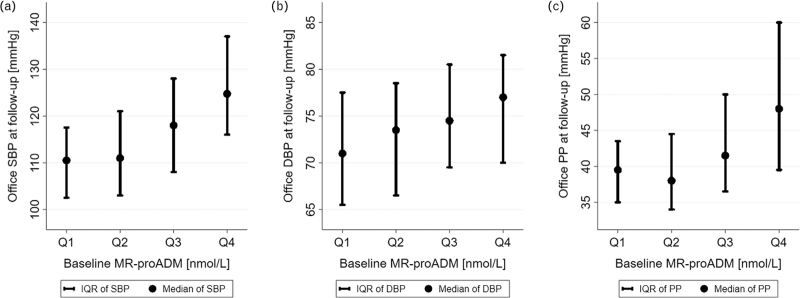
Unadjusted levels of systolic blood pressure (a), diastolic blood pressure (b) and pulse pressure (c) at 3-year follow-up per quartile of baseline mid-regional pro-adrenomedullin levels at baseline. Quartiles of baseline MR-proADM were defined as follows: Q1 = 0.227–0.388 nmol/l; Q2 = 0.389–0.454 nmol/l; Q3 = 0.455–0.532 nmol/l; Q4 = 0.533–1.544 nmol/l. SBP, systolic blood pressure; DBP, diastolic blood pressure; PP, pulse pressure; MR-proADM, mid-regional pro-adrenomedullin.

SKIPOGH included 1128 participants, the present analysis included 843 subjects (Fig. 1), whose characteristics at baseline and follow-up are shown in Table 1. 51.1% were women; age ranged from 18 to 86 years at baseline. The mean (SD) follow-up time was 3 (0.4) years. Approximately a quarter were hypertensive at baseline, of which 20.4% reported taking BP-lowering medications. Prevalence of hypertension and use of BP-lowering treatment was slightly higher at follow-up. Median [IQR] difference in SBP, DBP, and PP between baseline and follow-up was −0.5 [−8; 7], −2 [−7; 4] and 1 [−5; 7] mmHg, respectively. The median [IQR] MR-proADM plasma concentration was 0.453 [0.387; 0.531] nmol/l at baseline and the total range went from 0.227 to 1.544 nmol/l. The median and interquartile range of SBP, DBP, and PP at follow-up per quartile of baseline MR-proADM are shown in Fig. 2. Differences between included and excluded participants are shown in Table S1, Supplemental Digital Content.

**TABLE 1:** Characteristics of participants at baseline and follow-up

Characteristics	Baseline (*N* = 843)	Follow-up (*N* = 843)
Age at clinical visit	49 [34; 62]	52 [37; 65]
Women, *n* (%)	431 (51.1%)	431 (51.1%)
Body mass index, kg/m^2^,	24.8 [21.7; 27.2]	25.15 [21.9; 27.5]
Current smoking, *n* (%)	190 (22.5%)	199 (23.8%)
Office systolic BP, mmHg	117 (16)	118 (16)
Office diastolic BP, mmHg	76 (9)	74 (9)
Office pulse pressure, mmHg	42 (12)	44 (12)
Hypertension, *n* (%)	223 (26.5%)	225 (26.7%)
Antihypertensive medication, *n* (%)	172 (20.4%)	177 (21.2%)
Diabetes, *n* (%)	35 (4.2%)	48 (5.7%)
Lipid-lowering medication, *n* (%)	95 (11%)	106 (13%)
Estimated glomerular filtration rate, ml/min/1.73 m^2^	96 (18)	92 (18)
Chronic kidney disease, *n* (%)	28 (3%)	34 (4%)
MR-proADM, nmol/l	0.453 [0.387; 0.531]	0.411 [0.323; 0.505]

Data are shown in mean (SD) or median [IQR] for continuous variables and in frequencies (%) for categorical variables. Hypertension was defined as systolic BP ≥140 mmHg or diastolic BP ≥90 mmHg and/or use of antihypertensive medication. Diabetes was defined as fasting blood glucose ≥7 mmol/l, use of hypoglycemic medication and/or self-reported diabetes in questionnaire. BP, blood pressure; MR-proADM, mid-regional pro-adrenomedullin.

### Cross-sectional BP analyses

In univariate analyses, MR-proADM was positively associated with SBP, DBP and PP, for both office and 24-h ABPM (Table S2, Supplemental Digital Content). No association was found with heart rate. Results of the multivariate models are presented in Table 2. Adjustment for age, and sex (Model 1) greatly reduced the amplitude of the β-coefficient for MR-proADM. After further adjusting for BMI, antihypertensive medication and eGFR, the association between MR-proADM and office, 24-h, daytime DBP became negative (Model 2). The negative association persisted even after further adjustment for classical cardiovascular risk factors (Model 3). Furthermore, we found a positive association between MR-proADM and nighttime SBP and nighttime PP. We found no association between MR-proADM and office, 24 h, daytime SBP.

**TABLE 2:** Association between MR-proADM and BP at baseline (*N* = 843)

	Model 1^a^		Model 2^b^		Model 3^c^	
Outcome	MR-proADM	*P*-value	MR-proADM	*P*-value	MR-proADM	*P*-value
Systolic BP						
24-h	8.9 (1.2, 16.7)	0.025	1.2 (−7.6, 10.0)	0.792	1.5 (−7.4, 10.4)	0.743
Daytime	5.6 (−2.5, 13.8)	0.176	−2.2 (−11.2, 7.2)	0.671	−1.5 (−10.9, 7.8)	0.746
**Nighttime**	**18.8 (10.5, 27.1)**	**<0.001**	**12.3 (2.8, 21.9)**	**0.011**	**12.3 (2.7, 21.9)**	**0.012**
Office	10.5 (1.5, 29.5)	0.022	−2.6 (−12.5, 7.3)	0.606	−2.0 (−12.0, 8.0)	0.696
Diastolic BP						
**24-h**	2.0 (−2.9, 6.8)	0.427	−**5.8 (**−**11.3,** −**0.3)**	**0.039**	−**5.9 (**−**11.5,** −**0.4)**	**0.036**
**Daytime**	1.4 (−4.0, 6.7)	0.618	−**6.8 (**−**12.8,** −**0.7)**	**0.029**	−**7.0 (**−**13.2,** −**0.8)**	**0.026**
Nighttime	3.3 (−1.7, 8.2)	0.195	−2.5 (−8.2, 3.2)	0.388	−2.6 (−8.3, 3.2)	0.382
**Office**	−0.02 (−5.6, 5.6)	0.994	−**10.8 (**−**16.9,** −**4.6)**	**0.001**	−**9.8 (**−**16.1,** −**3.6)**	**0.002**
Pulse pressure						
24-h	5.7 (0.2, 11.1)	0.042	4.6 (−1.7, 10.9)	0.152	4.8 (−1.6, 11.2)	0.143
Daytime	3.0 (−2.5, 8.6)	0.290	2.6 (−3.9, 9.1)	0.433	3.1 (−3.5, 9.7)	0.352
**Nighttime**	**12.8 (6.4, 19.2)**	**<0.001**	**10.5 (3.0, 17.9)**	**0.006**	**9.8 (2.3, 17.3)**	**0.011**
Office	7.6 (1.3, 14.0)	0.018	4.1 (−3.2, 11.3)	0.276	3.4 (−3.9, 10.8)	0.360

Values are β-coefficient (95% confidence intervals).

a Model 1: adjusted for age, sex, study center; further adjusted for age squared for diastolic and pulse pressure.

b Model 2: further adjuster for body mass index, estimated glomerular filtration rate, BP-lowering treatment.

c Model 3: further adjuster for lipid-lowering treatment, presence of diabetes, smoking status, units of alcohol per week. BP, blood pressure; MR-proADM, mid-regional pro-adrenomedullin; PP, pulse pressure.

### Longitudinal BP analyses

In univariate analyses, baseline MR-proADM was not associated with ΔSBP, ΔDBP or ΔPP (Table S3, Supplemental Digital Content). Results of multivariable regressions are shown in Table 3. No associations were found in Model 1. In Model 2, MR-proADM was positively associated only with changes in PP. In the fully adjusted model (Model 3), MR-proADM was associated with an increase in SBP [β (95% CI): 8.2 (0.4, 15.9)], and an increase in PP [β (95% CI): 6.4 (0.3, 12.4)].

**TABLE 3:** Association between baseline MR-proADM and changes in BP at 3-year follow-up (*N* = 843)

	Model 1^a^		Model 2^b^		Model 3^c^	
Outcome	MR-proADM	*P*-value	MR-proADM	*P*-value	MR-proADM	*P*-value
ΔSBP	3.1 (−4.7, 12.0)	0.435	6.8 (−0.8, 14.5)	0.081	**8.2 (0.4, 15.9)**	**0.039**
ΔDBP	0.3 (−4.9, 5.6)	0.896	−2.0 (−7.0, 3.1)	0.444	−1.4 (−6.5, 3.7)	0.590
ΔPP	1.4 (−4.6, 7.4)	0.650	**6.0 (0.2, 11.9)**	**0.049**	**6.4 (0.3, 12.4)**	**0.039**

Values are β-coefficient (95% confidence intervals).

a Model 1: adjusted for baseline age, sex, study center; further adjusted for age squared for diastolic and pulse pressure.

b Model 2: further adjuster for baseline BP (SBP, DBP or PP for models with ΔSBP, ΔDBP, ΔPP as outcomes, respectively), baseline body mass index, changes in weight at follow-up, presence of chronic kidney disease at baseline, changes in estimated glomerular filtration at follow-up, use of BP-lowering treatment at baseline.

c Model 3: further adjuster for lipid-lowering treatment at baseline, presence of diabetes at baseline, smoking status at baseline, units of alcohol per week at baseline. BP, blood pressure, ΔSBP, changes in systolic BP; ΔDBP, changes in diastolic BP; ΔPP, changes in pulse pressure; MR-proADM, mid-regional pro-adrenomedullin.

The most important confounding variables in our model were age, sex and BMI (Tables S9−S11, Supplemental Digital Content). Interaction between MR-proADM and sex for their association with BP was not statistically significant. Introduction of menopausal status as a covariable instead of sex (coded as follows: 0, men; 1, premenopausal women; 3, postmenopausal women), did not improve the model and the variable did not reach statistical significance. Presented models were not adjusted for prior CVD as we believed this may lead to collider bias. However, we tried to introduce this variable into our models, this didn’t change our conclusions (data not shown). ADM has been shown to inhibit aldosterone agonist stimulated secretion in vitro. In human studies, decreased plasma aldosterone concentration following ADM has been shown in individuals with chronic and acute heart failure. In healthy individuals, ADM increases plasma renin activity but not plasma aldosterone concentration [13]. Addition of aldosterone to the models did not change our conclusion and did not reach statistical significance. We therefore decided to exclude it from our models. Addition of plasma renin activity did reach statistical significance but did not change our conclusion (data not shown)

Sensitivity analyses were conducted excluding participants with (a) hypertension and (b) both hypertension and diabetes. This reinforced the association (Tables S4 and S5, Supplemental Digital Content). Exclusion of individuals with untreated hypertension at baseline and individuals that started or stopped antihypertensive medication during follow-up did not change our conclusions (Tables S6 and S7, Supplemental Digital Content).

### Longitudinal arterial stiffness analyses

We found no association between MR-proADM and central SBP, DBP, or PP. Although it should be noted that effect size was similar between models with office and central SBP as well as PP and sample size was smaller (*N* = 695) (Table 4).

**TABLE 4:** Association between baseline MR-proADM and changes in central BP at 3-year follow-up

	*N*	Model 1^a^		Model 2^b^		Model 3^c^	
Outcome		MR-proADM	*P*-value	MR-proADM	*P*-value	MR-proADM	*P*-value
Δ central SBP	695	1.2 (−9.6, 11.9)	0.831	7.1 (−3.2, 17.4)	0.178	8.5 (−2.6, 19.6)	0.132
Δ central DBP	695	−1.4 (−8.2, 5.3)	0.677	0.2 (−6.5, 6.8)	0.958	1.5 (−5.7, 8.7)	0.680
Δ central PP	695	3.0 (−5.8, 11.7)	0.508	5.1 (−3.4, 13.6)	0.238	5.2 (−3.9, 14.3)	0.263
ΔPWV	649	2.5 (−0.01, 5.1)	0.051	0.8 (−0.3, 1.9)	0.171	0.8 (−0.4, 2.0)	0.212
ΔAI	628	4.7 (−7.1, 16.6)	0.434	7.8 (−3.9, 19.4)	0.192	5.3 (−7.5, 18.1)	0.415
ΔRRI	509	−0.01 (−0.1, 0.02)	0.449	0.01 (−0.03, 0.05)	0.679	0.01 (−0.03, 0.05)	0.500

Values are β-coefficient (95% confidence intervals).

a Model 1: adjusted for baseline age, sex, study center; further adjusted for age squared for diastolic and pulse pressure.

b Model 2: further adjusted for baseline central BP (central SBP, central DBP or central PP for models with Δ central SBP, Δ central DBP, Δ central PP as outcomes, respectively), PWV or AI, baseline BMI, changes in weight at follow-up, presence of chronic kidney disease at baseline, changes in estimated glomerular filtration rate at follow-up, use of BP-lowering treatment at baseline.

c Model 3: further adjuster for lipid-lowering treatment at baseline, presence of diabetes at baseline, smoking status at baseline, units of alcohol per week at baseline. Models 2 and 3 were further adjusted for baseline SBP, DBP and heart rate for PWV, AI and RRI. BP, blood pressure, Δ central SBP, changes in central systolic BP; ΔDBP, changes in central diastolic BP; ΔPP, changes in central pulse pressure; ΔPWV, changes in pulse wave; ΔAI, changes in augmentation index; ΔRRI, changes in renal resistive index; MR-proADM, mid-regional pro-adrenomedullin.

MR-proADM was neither associated with PWV, nor with AI, or RRI (Table 4).

## DISCUSSION

In the present analyses, baseline MR-proADM was negatively associated with baseline DBP (office, 24-h and daytime). On the contrary, baseline MR-proADM was positively associated with baseline nighttime SBP and PP. In longitudinal analyses, baseline MR-proADM levels were associated with an increase in office SBP and PP at 3-year follow-up, and not with DBP. While the cross-sectional analyses are in line with the well known vasodilatory properties of ADM, the longitudinal analyses confirm prior findings suggesting that ADM levels could represent a compensatory mechanism of elevated BP, which could be used to predict future risk of developing arterial hypertension and/or arterial stiffness [23].

In line with our results, Yucel *et al.* reported a positive association between MR-pro-ADM and nighttime SBP in participants with newly diagnosed hypertension [22]. In contrast, Kario *et al.*[29], found no association between ABPM and ADM in elderly hypertensive participants. This may be explained by confounding factors as results were unadjusted. In our study mere adjustment for age and sex considerably changed the association, suggesting that important confounders exist between BP and ADM. As hypothesized by Yucel *et al.*, the association between MR-proADM and nighttime SBP may mark an underlying endothelial dysfunction. Associations between nondipping pattern, nocturnal hypertension and endothelial dysfunction have been previously described. In a study conducted on hypertensive inpatients, nondippers displayed impaired endothelium-dependent vasodilation through a decrease in NO release compared to dippers [42]. It should be noted that nighttime SBP is the ABPM variable that best correlates with cardiovascular morbidity and mortality [43–45]. ADM induces vasodilatation by increasing endothelial NO synthase [1,2]. Increased ADM in nondippers may reflect impaired NO release.

We found a negative association between MR-proADM and baseline office, 24-h, and daytime DBP. ADM's vasodilator effect is well documented in human studies. In line with our results, in a randomized control cross-over study administration of continuous intravenous ADM infusion decreased DBP (5 mmHg) but not SBP [46]. The most obvious explanation of the more pronounced reduction of DBP rather than SBP is ADM's vasodilator effect as greater ADM levels were associated with a lower diastolic BP. This is most likely due to an ADM-induced reduction of peripheral vascular resistance. The reduced peripheral resistance could in turn lead to an increased cardiac output and hence SBP. Nevertheless, in studies like ours, it is difficult to dissociate an increased cardiac output due to an ADM-induced decrease in peripheral vascular resistances from an ADM-induced inotropic effect affecting SBP. To clarify the specific impact of each of these mechanisms, specific physiological studies are needed. Other studies have reported a significant decrease in both SBP and DBP during intravenous ADM administration (from 3 to 25 mmHg depending on the dose and study) [9,10,47]. Regarding cross-sectional observational studies, discrepant results have been published concerning the association between (MR-pro)ADM and office SBP, DBP, PP. Two Japanese studies found a positive association between MR-proADM and both SBP and DBP among hypertensive participants as well as in elderly and middle-aged individuals [30,31]. Al-Omari *et al.*[18] reported a positive association with SBP and negative with DBP in African Americans with hypertension in the Genetic Epidemiology Network of Arteriopathy (GENOA) study and some found no correlation in the general population in Japan [24]. Positive associations with PP were reported in the general population in Japan, in African Americans and non-Hispanic whites in the GENOA study and in middle-aged individuals in Sweden in the Mälmo Diet Cancer Study [24,26,27].

It should be noted that cross-sectional analysis using linear regression in absence of instrumental variables is problematic when studying the association between BP and MR-proADM. ADM has been established as a vasodilator. ADM levels are increased in hypertensive individuals, most likely because ADM release is activated by the inflammation and increased shear stress. This bidirectional association creates a simultaneity problem, violating a fundamental assumption of linear regressions, leading to potentially bias results. We hypothesized that examining the association in a longitudinal manner eliminates this issue as baseline MR-proADM should not be able to directly affect BP several years later. However, the mechanisms may be more complex as circulating levels of MR-proADM may not adequately reflect the total body effect of ADM on BP. Indeed, ADM is suspected of having paracrine and autocrine actions, which may not be adequately captured by circulating ADM levels.

To the best of our knowledge, this is the second cohort study to examine the longitudinal association between ADM and continuous SBP [35] and the first to report an association with PP. In our study, MR-proADM was positively associated with changes in SBP and PP over a mean follow-up of three years, although it should be noted that confidence intervals were rather large. Sensitivity analyses showed that this association was strengthened when excluding hypertensive and diabetic participants. These results suggest that ADM may be useful marker to predict development of hypertension and/or arterial rigidity.

In contrast to our findings, Ohlsson *et al.* reported a negative association between baseline MR-proADM and ΔSBP [35]. We replicated Ohlsson *et al.*'s model and found a positive association (Table S8, Supplemental Digital Content), consistent with our results. This discrepancy may be explained by our much shorter follow-up time (3 years vs. 17 years) or our smaller sample size. It could also be explained by analytic consideration. MR-proADM was measure several years after collection, so possible measurement errors could arise (samples were collected from 1991 to 1996 [35], MR-proADM was measured in 2008 in plasma frozen at −80°C).

Kato *et al.* examined the association between baseline ADM and incidence of hypertension. After separating their cohort into two, they found significantly higher rates of hypertension in the high-ADM group. However, in multivariable analysis, risk of development of hypertension at 3-year follow-up was not associated with baseline ADM [23]. This could be explained by short follow-up time or insufficiently accurate measures of ADM when used as a continuous variable. In fact, the group used ADM instead of MR-proADM, which has a short half-life of 20–25 min [2].

As an association between baseline MR-proADM and 3-year changes in PP was found, we sought to explore whether this association was also true for direct indicators of arterial stiffness. We found no association between MR-proADM and PWV, RRI or AI. This may be explained by a short follow-up time of 3-years or insufficient power. Alternatively, the association between MR-proADM and PP may simply be another expression of the association between MR-proADM and SBP, as PP is the difference between SBP and DBP.

Our study has some limitations. First, follow-up time was short, so we were unable to study the association between MR-proADM and incidence of hypertension. Second, the study had limited power and the sample was exclusively composed of individuals of European descend, which limits its generalizability. Finally, as MR-proADM remains an under-studied parameter, residual confounding cannot be excluded. Our study also has some strengths: it is a population-based and multicentric cohort study, and we measured MR-proADM levels, which is a more accurate measure of circulating ADM.

## CONCLUSION

Baseline MR-proADM levels were positively associated with changes in SBP and PP over a mean follow-up of three years. Further studies are required to clarify this association. These results suggest that ADM could be used to predict increases of BP over time, but it should be noted that confidence intervals were rather large. Studies with even larger study samples and longer follow-up times are needed to explore whether a score to predict development of hypertension using ADM as one of the covariables could be clinically relevant.

## ACKNOWLEDGEMENTS

We thank the study nurses involved in the study and recruitment: Marie-Odile Levy, Guler Gök-Sogüt, Ulla Spüchbach, Dominique Siminski. We thank Sandrine Estoppey and Dusan Petrovic for their help in logistics and database management. We are grateful to the SKIPOGH study participants.

All authors had full access to all the data in the study and take responsibility for its integrity and the data analysis.

Full work presented at: Symposium Rhône-Alpes, Hypertension research Foundation, Switzerland.

Funding: This work was supported financially by a grant from the Swiss National Science Foundation (FN 33CM30-124087).

### Conflicts of interest

Authors have no conflict of interest to declare.

## Supplementary Material

Supplemental Digital Content
