# Supplementary material for: Association of mid-regional pro-adrenomedullin with office and 24-h ambulatory blood pressure in a Swiss general population sample
Source: J Hypertens. 2024 Sep 18;42(12):2187–95. doi: 10.1097/HJH.0000000000003866 (PMC11556881; doi:10.1097/HJH.0000000000003866)
Supplement: Supplemental Digital Content [file jhype-42-2187-s001.doc]

| **Table S1 – Comparison of characteristics between included and excluded participants** | | | |
| --- | --- | --- | --- |
| **Characteristics** | **Included (N=843)** | **Excluded (N=336)** | **P-value** |
| Age at clinical visit | 49 [34; 62] | 45 [30; 60] | 0.062^a^ |
| Women, n (%) | 431 (51.1%) | 187 (55.6%) | 0.160^a^ |
| Body mass index, kg/m^2^, | 24.8 [21.7; 27.2] | 25.51 [22.1; 28.3] | 0.102^b^ |
| Current smoking, n (%) | 190 (22.5%) | 82 (29.5%) | 0.019^c^ |
| Office systolic BP, mmHg | 117 (16) | 117 (18) | 0.443^a^ |
| Office diastolic BP, mmHg | 76 (9) | 75 (10) | 0.716^a^ |
| Office pulse pressure, mmHg | 42 (12) | 42 (14) | 0.451^a^ |
| Hypertensive, n (%) | 223 (26.5%) | 71 (25.0%) | 0.233^c^ |
| Antihypertensive medication, n (%) | 172 (20.4%) | 51 (18.1%) | 0.715^c^ |
| Diabetes, n (%) | 35 (4.2%) | 27 (9.3%) | 0.018^c^ |
| Lipid-lowering medication, n (%) | 95 (11%) | 23 (8%) | 0.140^c^ |
| eGFR, ml/min/1.73m^2^ | 96 (18) | 95 (18) | 0.091^a^ |
| Chronic kidney disease, n (%) | 28 (3%) | 7 (2%) | 0.258^c^ |
| MR-proADM, nmol/L | 0.453 [0.387; 0.531] | 0.450 [0.386; 0.536] | 0.835^b^ |
| Data are shown in mean (SD) or median [IQR] for continuous variables and in frequencies (%) for categorical variables.  Differences between included and excluded participants we analyzed using t-test^a^ for normally distributed data, Mann-Whitney tests^b^ for non-normally distributed data and chi-square tests^c^ for categorical variables.  Hypertension was defined as systolic BP ≥ 140 or diastolic BP ≥ 90 mmHg and/or use of anti-hypertensive medication.  Diabetes was defined as fasting blood glucose ≥ 7 mmol/L, use of hypoglycemic medication and/or self-reported diabetes in questionnaire.  BP indicates blood pressure; MR-proADM, mid-regional pro-adrenomedullin; eGFR, Estimated Glomerular Filtration Rate. | | | |

| **Table S2 – Univariable association between BP indicators and MR-pro-ADM: cross-sectional analysis (N=843)** | | |
| --- | --- | --- |
| **Outcome** | **MR-pro-ADM** | **P-value** |
| Systolic BP |  |  |
| 24h | 23.5 (16.9, 30.0) | 2.6∙10^-12^ |
| Daytime | 22.3 (15.3, 29.2) | 2.6∙10^-10^ |
| Nighttime | 26.6 (19.7, 33.5) | 3.8∙10^-14^ |
| Office | 46.4 (38.5, 54.3) | 4.7∙10^-31^ |
| Diastolic BP |  |  |
| 24h | 9.5 (5.2, 13.7) | 1.1∙10^-5^ |
| Daytime | 8.9 (4.3, 13.6) | 1.5∙10^-4^ |
| Nighttime | 11.1 (7.0, 15.2) | 1.1∙10^-7^ |
| Office | 12.1 (7.3, 16.9) | 7.4∙10^-7^ |
| Pulse Pressure |  |  |
| 24h | 13.5 (9.0, 18.0) | 3.7∙10^-9^ |
| Daytime | 12.3 (7.6, 16.9) | 7.6∙10^-7^ |
| Nighttime | 15.8 (10.6, 21.0) | 3.4∙10^-9^ |
| Office | 34.1 (28.4, 39.7) | 4.4∙10^-32^ |
| Values are β-coefficient (95% confidence intervals).  BP indicates Blood Pressure; MR-proADM, Mid-regional pro-adrenomedullin. | | |

| **Table S3 – Univariable association between follow-up BP indicators and baseline MR-pro-ADM: longitudinal analysis (N=843)** | | |
| --- | --- | --- |
| **Outcome** | **MR-pro-ADM** | **P-value** |
| Systolic BP | 1.7 (-4.6, 8.04) | 0.598 |
| Diastolic BP | -1.5 (-5.7, 2.8) | 0.502 |
| PP | 3.2 (-1.8, 8.3) | 0.211 |
| Values are β-coefficient (95% confidence intervals).  BP indicates Blood Pressure; MR-proADM, Mid-regional pro-adrenomedullin. | | |

| **Table S4: Association between of changes in blood pressure (**Δ**BP) indicators and MR-pro-ADM: longitudinal analysis, excluding hypertensive participants at baseline (N=620)** | | | | | | |
| --- | --- | --- | --- | --- | --- | --- |
|  | **Model 1^a^** | | **Model 2^b^** | | **Model 3^c^** | |
| **Outcome** | **MR-pro-ADM** | **P-value** | **MR-pro-ADM** | **P-value** | **MR-pro-ADM** | **P-value** |
| ΔSBP | 10.7 (1.4, 19.9) | 0.024 | 12.4 (3.6, 21.3) | 0.006 | 9.8 (0.7, 18.8) | 0.035 |
| ΔDBP | 0.7 (-6.5, 7.9) | 0.841 | -0.4 (-6.8, 6.0) | 0.899 | 0.1 (-6.3, 6.6) | 0.969 |
| ΔPP | 8.8 (0.8, 16.7) | 0.031 | 7.9 (0.2, 15.6) | 0.045 | 8.4 (0.6, 16.2) | 0.036 |
| Values are β-coefficient (95% confidence intervals).  ^a^Model 1: adjusted for baseline age, sex, study center; further adjusted for age squared for diastolic and pulse pressure.  ^b^Model 2: further adjuster for baseline BP (SBP, DBP or PP for models with ΔSBP, ΔDBP, ΔPP as outcomes, respectively), baseline body mass index, changes in weight at follow-up, presence of chronic kidney disease at baseline, changes in estimated glomerular filtration at follow-up.  ^c^Model 3: further adjuster for lipid-lowering treatment at baseline, presence of diabetes at baseline, smoking status at baseline, units of alcohol per week at baseline.  BP indicates blood pressure, ΔSBP indicates changes in systolic BP; ΔDBP, changes in diastolic BP; ΔPP, changes in pulse pressure; MR-proADM, Mid-regional pro-adrenomedullin. | | | | | | |

| **Table S5: Association between of changes in blood pressure (**Δ**BP) indicators and MR-pro-ADM: longitudinal analysis, excluding hypertensive participants and diabetic at baseline (N=607)** | | | | | | |
| --- | --- | --- | --- | --- | --- | --- |
|  | **Model 1^a^** | | **Model 2^b^** | | **Model 3^c^** | |
| **Outcome** | **MR-pro-ADM** | **P-value** | **MR-pro-ADM** | **P-value** | **MR-pro-ADM** | **P-value** |
| ΔSBP | 10.7 (1.4, 20.0) | 0.025 | 12.7 (3.7, 21.7) | 0.005 | 10.2 (1.0, 19.4) | 0.031 |
| ΔDBP | 0.6 (-6.7, 8.0) | 0.864 | -0.6 (-7.1, 5.8) | 0.850 | 0.1 (-6.4, 6.7) | 0.972 |
| ΔPP | 8.4 (0.3, 16.5) | 0.041 | 8.0 (0.2, 15.7) | 0.045 | 8.8 (0.9, 16.7) | 0.029 |
| Values are β-coefficient (95% confidence intervals).  ^a^Model 1: adjusted for baseline age, sex, study center; further adjusted for age squared for diastolic and pulse pressure.  ^b^Model 2: further adjuster for baseline BP (SBP, DBP or PP for models with ΔSBP, ΔDBP, ΔPP as outcomes, respectively), baseline body mass index, changes in weight at follow-up, presence of chronic kidney disease at baseline, changes in estimated glomerular filtration at follow-up.  ^c^Model 3: further adjuster for lipid-lowering treatment at baseline, smoking status at baseline, units of alcohol per week at baseline.  BP indicates blood pressure, ΔSBP indicates changes in systolic BP; ΔDBP, changes in diastolic BP; ΔPP, changes in pulse pressure; MR-proADM, Mid-regional pro-adrenomedullin. | | | | | | |

| **Table S6: Association between of changes in blood pressure (**Δ**BP) indicators and MR-pro-ADM: longitudinal analysis, excluding untreated hypertensive participants at baseline (N=792)** | | | | | | |
| --- | --- | --- | --- | --- | --- | --- |
|  | **Model 1^a^** | | **Model 2^b^** | | **Model 3^c^** | |
| **Outcome** | **MR-proADM** | **P-value** | **MR-proADM** | **P-value** | **MR-proADM** | **P-value** |
| ΔSBP | 3.5 (-4.4, 11.3) | 0.387 | 6.9 (-0.7, 14.5) | 0.076 | 8.1 (0.4, 15.7) | 0.038 |
| ΔDBP | -0.2 (-5.5, 5.1) | 0.940 | -3.5 (-8.5, 1.5) | 0.169 | -2.9 (-8.0, 2.1) | 0.249 |
| ΔPP | 1.5 (-4.7, 7.6) | 0.643 | 6.8 (0.7, 12.9) | 0.028 | 7.2 (1.0, 13.3) | 0.022 |
| Values are β-coefficient (95% confidence intervals).  ^a^Model 1: adjusted for baseline age, sex, study center; further adjusted for age squared for diastolic and pulse pressure.  ^b^Model 2: further adjuster for baseline BP (SBP, DBP or PP for models with ΔSBP, ΔDBP, ΔPP as outcomes, respectively), baseline body mass index, changes in weight at follow-up, presence of chronic kidney disease at baseline, changes in estimated glomerular filtration at follow-up, use of BP-lowering treatment at baseline.  ^c^Model 3: further adjuster for lipid-lowering treatment at baseline, presence of diabetes at baseline, smoking status at baseline, units of alcohol per week at baseline.  BP indicates blood pressure, ΔSBP indicates changes in systolic BP; ΔDBP, changes in diastolic BP; ΔPP, changes in pulse pressure; MR-proADM, Mid-regional pro-adrenomedullin. | | | | | | |

| **Table S7: Association between of changes in blood pressure (**Δ**BP) indicators and MR-pro-ADM: longitudinal analysis, excluding participants that started or stopped antihypertensive medication during follow-up (N=788)** | | | | | | |
| --- | --- | --- | --- | --- | --- | --- |
|  | **Model 1^a^** | | **Model 2^b^** | | **Model 3^c^** | |
| **Outcome** | **MR-proADM** | **P-value** | **MR-proADM** | **P-value** | **MR-proADM** | **P-value** |
| ΔSBP | 3.1 (-4.7, 11.0) | 0.435 | 6.9 (-0.8, 14.5) | 0.077 | **8.0 (0.4, 15.7)** | **0.040** |
| ΔDBP | 0.3 (-4.9, 5.6) | 0.896 | -2.4 (-7.4, 2.6) | 0.351 | -1.8 (-6.9, 3.2) | 0.475 |
| ΔPP | 1.4 (-4.6, 7.4) | 0.650 | **6.2 (0.2 12.1)** | **0.041** | **6.6 (0.6, 12.6)** | **0.032** |
| Values are β-coefficient (95% confidence intervals).  ^a^Model 1: adjusted for baseline age, sex, study center; further adjusted for age squared for diastolic and pulse pressure.  ^b^Model 2: further adjuster for baseline BP (SBP, DBP or PP for models with ΔSBP, ΔDBP, ΔPP as outcomes, respectively), baseline body mass index, changes in weight at follow-up, presence of chronic kidney disease at baseline, changes in estimated glomerular filtration at follow-up, use of BP-lowering treatment at baseline.  ^c^Model 3: further adjuster for lipid-lowering treatment at baseline, presence of diabetes at baseline, smoking status at baseline, units of alcohol per week at baseline.  BP indicates blood pressure, ΔSBP indicates changes in systolic BP; ΔDBP, changes in diastolic BP; ΔPP, changes in pulse pressure; MR-proADM, Mid-regional pro-adrenomedullin. | | | | | | |

| **Table S8: Reproduction of multivariable model as described in Ohlsson *et al.*** | | | | | | |
| --- | --- | --- | --- | --- | --- | --- |
|  | **Excluding hypertensive participants at baseline** | | **Additionally adjusted for antihypertensive treatment at follow-up** | | **Additionally adjusted for numbers of antihypertensive medications at follow-up** | |
| **Outcome** | **MR-pro-ADM** | **P-value** | **MR-pro-ADM** | **P-value** | **MR-pro-ADM** | **P-value** |
| ΔSBP | 1.3 (0.2, 2.4) | 0.023 | 1.2 (0.2, 2.4) | 0.030 | 1.2 (0.09, 2.3) | 0.033 |
| Values are given for z-score of the logarithmic value of MR-proADM as per in Ohlsson et al.’s study.  Models are adjusted for baseline age, sex, smoking habits, glucose, systolic blood pressure, body mass index, estimated glomerular filtration rate, and follow-up time. | | | | | | |

| **Table S9: Association between of changes in systolic blood pressure (ΔSBP) indicators and MR-pro-ADM: longitudinal analysis, with β -coefficient for each variable** | | | | | | |
| --- | --- | --- | --- | --- | --- | --- |
|  | **Model 1^a^** | | **Model 2^b^** | | **Model 3^c^** | |
| **Outcome** | ΔSBP | P-value | ΔSBP | P-value | ΔSBPP | P-value |
| **MR-proADM** | 3.1 (-4.7 ; 11.0) | 0.435 | 6.8 (-0.8 ; 14.5) | 0.081 | 8.2 (0.4; 15.9) | 0.039 |
| **Age at baseline** | -0.02 (-0.08; -0.04) | 0.549 | 0.2 (0.1; 0.2) | <0.001 | 0.2 (0.1; 0.2) | <0.001 |
| **Sex** | 1.3 (-0.3; 2.9) | 0.102 | 3.7 (2.3; 5.2) | <0.001 | 4.1 (2.6; 5.6) | <0.001 |
| **Study center**  **Geneva** | -1.5 (-3.5; 0.5) | 0.135 | -1.9 (-2.5; -0.3) | 0.020 | -1.9 (-3.5; -0.3) | 0.017 |
| **Bern** | 4.9 (2.6; 7.2) | <0.001 | 3.4 (1.5; 5.3) | <0.001 | 3.4 (1.5; 5.3) | <0.001 |
| **Baseline SBP** |  |  | -0.5 (-0.5; -0.4) | <0.001 | -0.4 (-0.5, -0.4) | <0.001 |
| **BMI at baseline** |  |  | 0.1 (-0.06; 0.3) | 0.168 | 0.1 (-0.1; 0.3) | 0.190 |
| **Weight gain** |  |  | 0.5 (0.3; 0.6) | <0.001 | 0.5 (0.3; 0.6) | <0.001 |
| **CKD category** |  |  | -0.3 (-4.6; 4.0) | 0.898 | -0.8 (-5.1; 3.5) | 0.724 |
| **CKD difference** |  |  | 0.06 (-0.01; 0.1) | 0.108 | 0.1 (-0.01, 0.1) | 0.092 |
| **Treated for hypertension (Yes; No)** |  |  | 0.01 (-2.0; 2.1) | 0.990 | -0.8 (-2.1, 0.6) | 0.265 |
| **Treated for hypercholesterolemia (Yes; No)** |  |  |  |  | 0.3 (-1.9; 2.4) | 0.757 |
| **Diabetic (Yes; No)** |  |  |  |  | -1.8 (-5.5; 1.9) | 0.349 |
| **Active smoker at baseline (Yes; No)** |  |  |  |  | 2.0 (0.3; 3.7) | 0.019 |
| **Units of alcohol consumed per week at baseline** |  |  |  |  | -0.003 (-0.04; 0.04) | 0.886 |
| Values are β-coefficient (95% confidence intervals).  ^a^Model 1: adjusted for baseline age, sex, study center; further adjusted for age squared for diastolic and pulse pressure.  ^b^Model 2: further adjuster for baseline DBP, baseline body mass index, changes in weight at follow-up, presence of chronic kidney disease at baseline, changes in estimated glomerular filtration at follow-up, use of BP-lowering treatment at baseline.  ^c^Model 3: further adjuster for lipid-lowering treatment at baseline, presence of diabetes at baseline, smoking status at baseline, units of alcohol per week at baseline.  BP indicates blood pressure, ΔDBP indicates changes in diastolic BP; MR-proADM, Mid-regional pro-adrenomedullin. | | | | | | |

| **Table S10: Association between of changes in diastolic blood pressure (ΔDBP) indicators and MR-pro-ADM: longitudinal analysis, with β -coefficient for each variable** | | | | | | |
| --- | --- | --- | --- | --- | --- | --- |
|  | **Model 1^a^** | | **Model 2^b^** | | **Model 3^c^** | |
| **Outcome** | ΔDBP | P-value | ΔDBP | P-value | ΔDBP | P-value |
| **MR-proADM** | 0.3 (-4.9 ; 5.6) | 0.896 | -2.0 (-7.0 ; 3.1) | 0.444 | -1.4 (-6.5; 3.7) | 0.590 |
| **Age at baseline** | -0.2 (-0.4; -0.02) | 0.029 | 0.1 (-0.1; 0.3) | 0.228 | 0.1 (-0.06; 0.3) | 0.233 |
| **Age squared at baseline** | 0.002 (-0.0001; -0.003) | 0.069 | -0.003 (-0.002; 0.001) | 0.684 | -0.003 (-0.002; 0.001) | 0.707 |
| **Sex** | 0.7 (-0.3; 1.7) | 0.216 | 2.2 (1.3; 3.2) | <0.001 | 2.4 (1.4; 3.4) | <0.001 |
| **Study center**  **Geneva** | -4.4 (-5.6; -3.1) | <0.001 | -3.4 (-4.5; -2.1) | <0.001 | -3.3 (-4.4; -2.3) | <0.001 |
| **Bern** | -5.3 (-6.8; -3.8) | <0.001 | -3.3 (-1.6; -5.3) | <0.001 | -3.3 (-4.5; -2.1) | <0.001 |
| **Baseline DBP** |  |  | -0.5 (-0.5; -0.4) | <0.001 | -0.5 (-0.5, -0.4) | <0.001 |
| **BMI at baseline** |  |  | 0.3 (0.2; 0.4) | <0.001 | 0.3 (0.2; 0.4) | <0.001 |
| **Weight gain** |  |  | 0.4 (0.3; 0.5) | <0.001 | 0.4 (0.3; 0.5) | <0.001 |
| **CKD category** |  |  | 0.6 (-2.2; 3.3) | 0.699 | 0.2 (-2.6; 3.0) | 0.876 |
| **CKD difference** |  |  | 0.02 (-0.03; 0.1) | 0.442 | 0.02 (-0.03, 0.1) | 0.414 |
| **Treated for hypertension (Yes; No)** |  |  | -1.0 (-2.4; 0.3) | 0.122 | -0.8 (-2.1, 0.6) | 0.265 |
| **Treated for hypercholesterolemia (Yes; No)** |  |  |  |  | -0.7 (-2.3; 1.0) | 0.419 |
| **Diabetic (Yes; No)** |  |  |  |  | -1.7 (-4.1; 0.7) | 0.164 |
| **Active smoker at baseline (Yes; No)** |  |  |  |  | 0.8 (-0.3; 1.9) | 0.134 |
| **Units of alcohol consumed per week at baseline** |  |  |  |  | -0.001 (-0.03; 0.03) | 0.935 |
| Values are β-coefficient (95% confidence intervals).  ^a^Model 1: adjusted for baseline age, sex, study center; further adjusted for age squared for diastolic and pulse pressure.  ^b^Model 2: further adjuster for baseline DBP, baseline body mass index, changes in weight at follow-up, presence of chronic kidney disease at baseline, changes in estimated glomerular filtration at follow-up, use of BP-lowering treatment at baseline.  ^c^Model 3: further adjuster for lipid-lowering treatment at baseline, presence of diabetes at baseline, smoking status at baseline, units of alcohol per week at baseline.  BP indicates blood pressure, ΔDBP indicates changes in diastolic BP; MR-proADM, Mid-regional pro-adrenomedullin. | | | | | | |

| **Table S11: Association between of changes in pulse pressure (ΔPP) indicators and MR-pro-ADM: longitudinal analysis, with β-coefficient for each variable** | | | | | | |
| --- | --- | --- | --- | --- | --- | --- |
|  | **Model 1^a^** | | **Model 2^b^** | | **Model 3^c^** | |
| **Outcome** | ΔPP | P-value | ΔPP | P-value | ΔPP | P-value |
| **MR-proADM** | 1.4 (-4.6 ; 7.4) | 0.650 | 6.0 (0.02; 11.9) | 0.049 | 6.4 (0.3, 12.4) | 0.039 |
| **Age at baseline** | -0.05 (-0.3; 0.2) | 0.633 | -0.4 (-0.6; -0.2) | <0.001 | -0.4 (-0.6, -0.2) | <0.001 |
| **Age squared at baseline** | 0.001 (-0.0001; -0.003) | 0.492 | 0.006 (0.004; 0.01) | <0.001 | 0.006 (0.004; 0.008) | <0.001 |
| **Sex** | 0.6 (-0.6; 1.8) | 0.356 | 1.5 (0.4; 2.6) | 0.008 | 1.6 (0.4; 2.) | 0.0078 |
| **Study center**  **Geneva** | 3.0 (1.6; 4.5) | <0.001 | 1.5 (0.3; 2.7) | 0.018 | 1.5 (0.3; 2.) | 0.017 |
| **Bern** | 10.2 (8.6; 11.8) | <0.001 | 6.5 (5.1; 8.0) | <0.001 | 6.5 (5.1; 8.1) | <0.001 |
| **Baseline PP** |  |  | -0.5 (-0.5; -0.4) | <0.001 | -0.5 (-0.5, -0.4) | <0.001 |
| **BMI at baseline** |  |  | -0.02 (-0.2; 0.1) | 0.791 | 0.3 (0.2; 0.4) | <0.001 |
| **Weight gain** |  |  | 0.1 (-0.1; 0.2) | 0.353 | 0.4 (0.3; 0.5) | <0.001 |
| **CKD category** |  |  | -1.7 (-5.0; 1.6) | 0.317 | -1.9 (-5.2; 1.5) | 0.271 |
| **CKD difference** |  |  | 0.04 (-0.02; 0.1) | 0.159 | 0.04 (-0.01, 0.1) | 0.140 |
| **Treated for hypertension (Yes; No)** |  |  | -0.5 (-1.0; 2.1) | 0.495 | 0.7 (-0.1, 2.3) | 0.431 |
| **Treated for hypercholesterolemia (Yes; No)** |  |  |  |  | -0.3 (-2.2; 1.7) | 0.791 |
| **Diabetic (Yes; No)** |  |  |  |  | -0.8 (-0.5; 2.1) | 0.231 |
| **Active smoker at baseline (Yes; No)** |  |  |  |  | 0.8 (-0.5; 2.1) | 0.231 |
| **Units of alcohol consumed per week at baseline** |  |  |  |  | 0.008 (-0.02; 0.04) | 0.638 |
| Values are β-coefficient (95% confidence intervals).  ^a^Model 1: adjusted for baseline age, sex, study center; further adjusted for age squared for diastolic and pulse pressure.  ^b^Model 2: further adjuster for baseline PP, baseline body mass index, changes in weight at follow-up, presence of chronic kidney disease at baseline, changes in estimated glomerular filtration at follow-up, use of BP-lowering treatment at baseline.  ^c^Model 3: further adjuster for lipid-lowering treatment at baseline, presence of diabetes at baseline, smoking status at baseline, units of alcohol per week at baseline.  BP indicates blood pressure, ΔPP indicates changes in pulse pressure; MR-proADM, Mid-regional pro-adrenomedullin. | | | | | | |
